# Kinematic versus mechanical alignment: A systematic review of systematic reviews and meta‐analyses of randomised controlled trials

**DOI:** 10.1002/jeo2.70044

**Published:** 2024-10-30

**Authors:** Amir A. Jamali, Adithya Shekhar, Danton Dungy, Susan L. Stewart

**Affiliations:** ^1^ Joint Preservation Institute Walnut Creek California USA; ^2^ The Dungy Orthopedic Center Chandler Arizona USA; ^3^ Department of Public Health Sciences University of California Davis California USA; ^4^ Medical Sciences 1‐C, One Shields Avenue Davis California USA

**Keywords:** alignment, kinematic, knee osteoarthritis, total knee arthroplasty, total knee replacement

## Abstract

**Purpose:**

The purpose of this study was to review the currently available systematic reviews and meta‐analyses comparing kinematic alignment (KA) and mechanical alignment (MA).

**Methods:**

A literature search was performed to obtain all systematic review and meta‐analyses comparing KA to MA that included one or more randomised controlled trials. A total of 18 studies were obtained, three of which were systematic reviews without meta‐analysis. Studies were evaluated based on their meta‐analysis methodology, appropriate inclusion criteria, the use of correct definitions of each alignment technique, and risk of bias.

**Results:**

These 18 studies included between 3 and 14 RCTs in each study. From the perspective of study design, the majority of papers had low risk of bias. In contrast, most of these reviews had technical issues pertaining to study inclusion in their meta‐analyses that would potentially compromise their conclusions. These included mixing time points in the analysis, duplicate inclusion of patients in a meta‐analysis, inclusion of studies with incorrect definitions of KA, inclusion of studies performed with restricted kinematic alignment with the KA group, and inappropriate combination of studies with bilateral total knee arthroplasty (TKA) with studies with unilateral TKA.

**Conclusions:**

The current literature is inadequate to determine if there is any advantage to KA compared to MA in TKA. Claims made in systematic reviews and meta‐analyses on the subject must be carefully scrutinised based not only on risk of bias but also on the included study populations, the surgical methodology of each underlying study, and the authors' understanding of the definitions of each alignment technique.

**Level of Evidence:**

Level 1 based on this study being a systematic review with the inclusion of only systematic reviews and meta‐analyses of randomised controlled trials.

**Abbreviations**
KA: kinematic alignment
KSS: Knee Society Score
MA: mechanical alignment
RCT: randomised controlled trial
rKA: restricted kinematic alignment
TKA: total knee arthroplasty

## INTRODUCTION

In spite of the great success of total knee replacement, a subgroup of patients continues to be dissatisfied with the procedure. The rates of dissatisfaction have varied in the literature between 10% and 20% [3, 14, 15, 23, 27, 28]. One potential explanation for these results has been the concern about laxity or over‐tightening of the knee as a result of changes in the joint line and alignment between the native state and the post‐surgical state. This has been postulated to change the kinematic envelope of the knee leading to pain transmission from the capsule and ligaments of the joint [7]. One method to address this concern has been the development of the kinematic alignment technique to match the alignment and position of the total knee with each person's variable native alignment [9, 11, 12]. In order to evaluate the merits of the kinematic alignment (KA) technique relative to the mechanical alignment (MA) technique, a number of case series and randomised controlled trials have been performed. Due to the relatively small numbers of patients in these studies, several authors have performed systematic reviews and meta‐analyses to further explore the benefits of KA versus MA. Many surgeons place significant confidence in the findings of these types of studies in their decision‐making algorithm for the type of alignment to pursue while performing TKA and in their conversations with patients. The objective of this study is to perform a systematic review of previous systematic reviews and meta‐analyses of randomised controlled trials of KA versus MA and to evaluate these reviews based on their selection criteria, reporting criteria, methodology, and conclusions. We hypothesised that in spite of the efforts made by the investigators in producing these reviews, the orthopaedic community still has limited information about the potential advantages of one technique over the other.

## METHODS

The Preferred Reports Items for Systematic Reviews and Meta‐Analyses (PRISMA) were followed. Methods of the analysis and inclusion criteria were specified in advance and documented a PROSPERO registered protocol. “Comparison of kinematic and mechanical alignment in total knee replacement: A systematic review/meta‐analysis of randomised controlled trials and summary of previous systematic reviews”, registration number: CRD42021219365.

### Eligibility criteria

The studies included in this project were all systematic reviews of randomised controlled trials of KA to MA in TKA with or without a meta‐analysis. Reviews that concurrently included case series or nonrandomized trials were not excluded but only the data on RCTs was included in our analysis.

### Information sources

We performed electronic searches using the Embase, PubMed, Scopus and Cochrane databases from database inception to 23 April 2024. This portion was performed with collaboration of all the authors with the exception of SLS.

### Search

The Embase, Scopus, and PubMed databases were searched with the following search terms in all fields (title, keywords, abstract…) for two searches. Search #1 was for “alignment” AND “total knee arthroplasty” AND “kinematic” AND “mechanical”. Search #2 was for “alignment” AND “total knee replacement” AND “kinematic” AND “mechanical”. For the Cochrane database, due to a slightly different database search functionality, we utilised the search “kinematic total knee replacement”.

### Study selection

Inclusion criteria were systematic reviews with or without meta‐analysis comparing KA to MA for total knee replacement based on patient reported outcomes and with at least one randomised controlled trial included in their review [12, 30].

Duplicates were removed and additional search in the combined series of abstracts was performed in the reference management software package for the terms “systematic review” OR “meta‐analysis” in all fields.

Two senior authors (AJ and DD), both fellowship‐trained orthopaedic knee replacement surgeons, independently performed the study selection. Discrepancies between the two reviewers were minimal and were resolved by discussion to reach a consensus.

Exclusion criteria included data pertaining to non‐English language studies, studies that were focused on the results of restricted kinematic alignment (rKA), and any network meta‐analyses. The authors of this manuscript have previously published a systematic review and meta‐analysis on this topic and due to risk of bias, that publication was also excluded from this review.

Using these selection criteria, we identified eighteen systematic reviews that met all criteria. We tabulated and analysed the included RCTs with particular attention to any characteristics that would call into question their inclusion in a systematic review and meta‐analysis of KA versus MA.

#### Data collection process and data items

For systematic reviews, the following information was recorded:

1. Number of included RCTs.
2. Inclusion of studies with correct definitions of KA and MA.
3. Inclusion of small studies (less than 30 patients).
4. Inclusion of studies that mixed “restricted kinematic alignment” with “kinematic alignment”.
5. Inclusion of varying time points in the same meta‐analysis.
6. Inclusion of the same population sample twice in the same meta‐analysis.
7. Inclusion of derived or correlated scores in a meta‐analysis without appropriate discussion in the manuscript.
8. Inclusion of studies with bilateral surgery.

#### Risk of bias in individual systematic reviews

Risk of bias in systematic reviews was tabulated with the ROBIS tool [40].

#### Summary measures

The systematic reviews and meta‐analysis principal summary measures were restricted to clinical scores including the WOMAC [1], Oxford Knee Score [26], Knee Society Pain, Function, and Combined scores [33] specific to each time point from surgery.

## RESULTS

**Figure 1:**
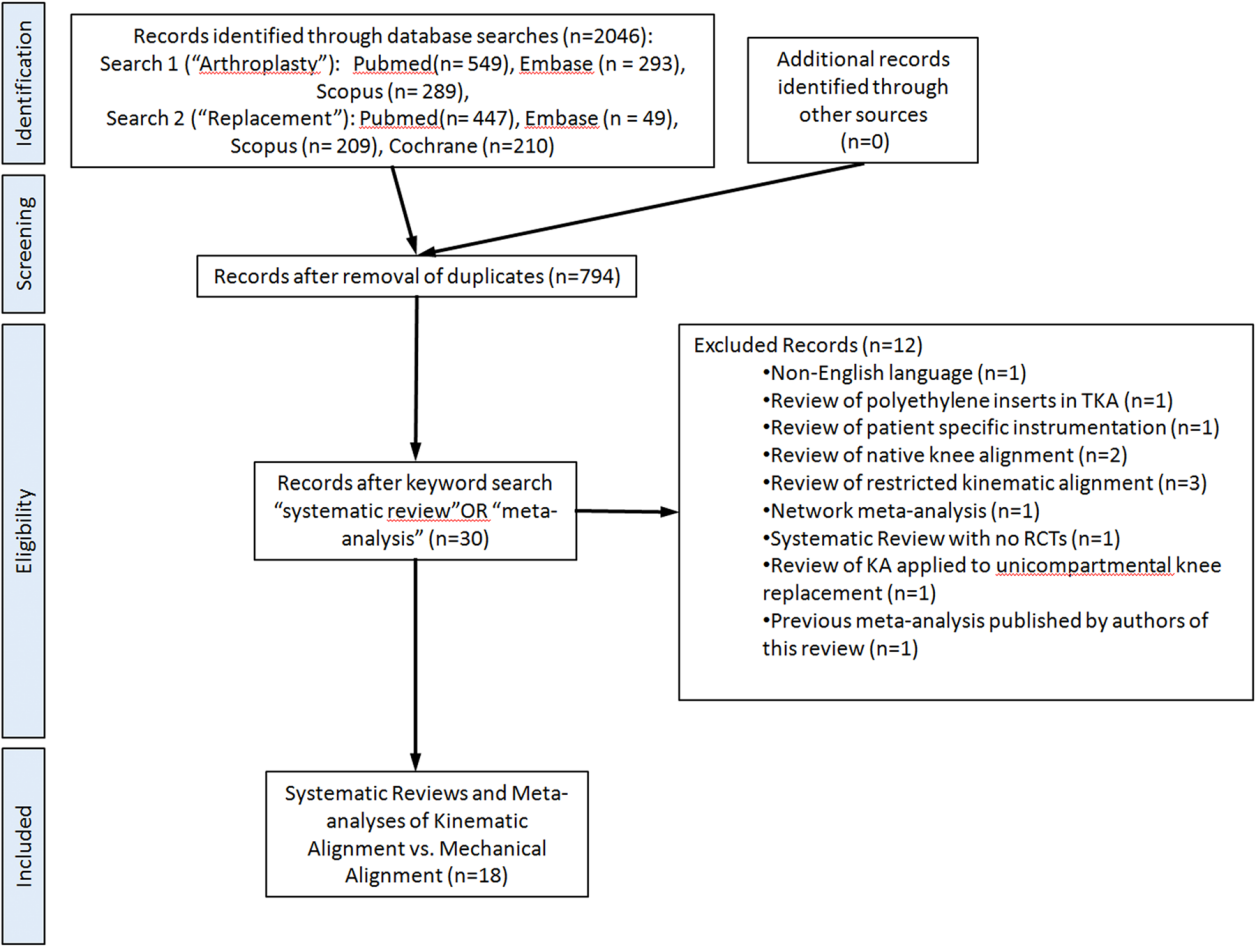
PRISMA flow diagram showing selection of the systematic reviews and meta‐analyses comparing KA and MA [19].

The total number of references for each database search were: Search #1: PubMed 549, Embase, 293 and Scopus 289 abstracts. Search #2: PubMed 447, Embase, 49 and Scopus 209 abstracts. The Cochrane search provided 210 abstracts. This led to a total of 2046 abstracts for review. 1252 abstracts were eliminated after removal of duplicates leaving 794 abstracts in the analysis. After further search of these abstracts in the reference management software for the terms “systematic review” or “meta‐analysis”, a total of 30 publications were obtained. Further review led to elimination of 12 of these. This left us with eighteen publications meeting our criteria of being a systematic review with or without meta‐analysis comparing KA to RA and inclusive of at least one randomised controlled trial. The PRISMA flow diagram showing the selection of these studies is shown in Figure 1.

The details of the eighteen included manuscripts are tabulated showing the included RCTs in each publication (Table 1) [4, 8, 10, 17, 18, 20, 21, 30, 31, 32, 35, 36, 37, 38, 39, 41, 42, 44]. Fourteen of the eighteen systematic reviews included at least one meta‐analysis. The reviews included between 3 and 14 reported RCTs.

**Table 1:** Study characteristics of 18 included systematic reviews/meta‐analyses of RCTs comparing KA and MA.

Authors	Title	Systematic Review	Meta‐analysis	Number of Included RCTs	Included RCTs														
					Dossett, 2012	Dossett, 2014	Belvedere, 2015	Calliess, 2017	Waterson, 2016	Young, 2016	Matsumoto, 2017	McNair, 2018	Yeo, 2019	Laende, 2019	McDessi, 2020	McEwen, 2020	Matsumoto, 2020	Young, 2020	Kaneda, 2022
Courtney et al.	Early outcomes of kinematic alignment in primary total knee arthroplasty: A meta‐analysis of the literature.	Yes	Yes	4		X		X	X	X									
Gao et al.	Comparison of kinematic alignment and mechanical alignment in total knee arthroplasty: A meta‐analysis of randomised controlled clinical trials.	Yes	Yes	11	X	X	X	X	X	X	X		X	X	X	X			
Hiyama et al.	Kinematically aligned total knee arthroplasty did not show superior patient‐reported outcome measures: An updated meta‐analysis of randomised controlled trials with at least 2‐year follow‐up.	Yes	Yes	4		X				X				X		X			
Lee et al.	Kinematic alignment is a possible alternative to mechanical alignment in total knee arthroplasty.	Yes	No	3		X		X		X									
Li et al.	Does kinematic alignment improve short‐term functional outcomes after total knee arthroplasty compared with mechanical alignment? A systematic review and meta‐analysis.	Yes	Yes	5	X	X		X	X	X									
Liu et al.	Kinematic alignment versus mechanical alignment in primary total knee arthroplasty: An updated meta‐analysis of randomised controlled trials.	Yes	Yes	14	X	X	X	X	X	X	X		X	X	X	X	X	X	X
Luo et al.	Similar results with kinematic and mechanical alignment applied in total knee arthroplasty.	Yes	Yes	6	X	X		X	X	X			X						
Riviere et al.	Alignment options for total knee arthroplasty: A systematic review.	Yes	No	5	X	X		X	X	X									
Roussot et al.	Clinical outcomes of kinematic alignment versus mechanical alignment in total knee arthroplasty: A systematic review.	Yes	No	7	X	X		X	X	X		X			X				
Sappey‐Marinier et al.	Kinematic versus mechanical alignment for primary total knee arthroplasty with minimum 2 years follow‐up: A systematic review.	Yes	No	5	X					X			X	X		X			
Takahashi et al.	Kinematically aligned total knee arthroplasty or mechanically aligned total knee arthroplasty.	Yes	Yes	5		X		X	X	X	X								
Tian et al.	Kinematic alignment versus mechanical alignment in total knee arthroplasty: An up‐to‐date meta‐analysis	Yes	Yes	11		X		X	X	X	X		X	X	X	X	X	X	
Van Essen et al.	Kinematic alignment results in clinically similar outcomes to mechanical alignment: Systematic review and meta‐analysis.	Yes	Yes	11	X	X		X	X	X	X		X	X		X	X	X	
Wang et al., J Ortho Sci.	Kinematic and mechanical alignments in total knee arthroplasty: A meta‐analysis with >/=1‐year follow‐up.	Yes	Yes	11		X		X	X	X	X			X	X	X	X	X	X
Wang et al., KSSTA	Superiority of kinematic alignment over mechanical alignment in total knee arthroplasty during medium‐ to long‐term follow‐up: A meta‐analysis and trial sequential analysis.	Yes	Yes	7	X	X				X		X		X		X		X	
Woon et al.	Outcome of kinematic alignment using patient‐specific instrumentation versus mechanical alignment in TKA: A meta‐analysis and subgroup analysis of randomised trials.	Yes	Yes	4		X		X	X	X									
Xu et al.	Kinematic versus mechanical alignment for primary total knee replacement: A systematic review and meta‐analysis.	Yes	Yes	7	X	X	X	X	X	X	X								
Yoon et al.	Comparison of kinematic and mechanical alignment techniques in primary total knee arthroplasty: A meta‐analysis.	Yes	Yes	5	X	X	X	X		X									

A further analysis of the included RCTs sheds detail on the inputs into these systematic reviews. There were several studies which were deemed to be of either limited utility for inclusion in a review or, in some cases, inappropriate to include in a review of KA versus MA for TKA. Some of the meta‐analysis mixed divergent time points in the same meta‐analysis. Doing so would compromise the conclusions drawn since the results of total knee replacements can vary based on the time point measured. Another set of issues were related to reviews that included the same patient sample twice in the same meta‐analysis, thus skewing the results of these analyses. This was particularly true for two RCTs which reported explicitly on the same patient sample at two different follow‐up periods [5, 6, 45, 46]. Some reviews included studies which included small numbers of patients. For example, Belvedere et al. is an abstract comparing 11 patients with MA with 6 patients using KA [2]. The only clinical data presented was the IKDC score on this small group of patients. Along the same lines, the study by Kaneda is a small study of 13 knees in 9 patients comparing 8 TKAs with KA to 5 knees treated with MA [16]. Although they met the inclusion criteria in the systematic reviews in which they were included, in our opinion, these studies are not appropriate for inclusion in a meta‐analysis. MacDessi et al. is an RCT comparing MA not to KA but actually to a restricted kinematic alignment protocol (rKA) [22]. In the rKA technique, native alignment of the implants is matched to that of the patient's anatomy but a restriction is placed on the upper limits of an acceptable alignment within a set range. Patients whose alignment lies outside this set range are corrected to the outer bounds of the arbitrarily decided value in the protocol. Regardless of the theoretical advantages of KA to rKA, these two alignment techniques are clearly not the same technique and should not be combined into the same meta‐analysis. McEwen et al. published a RCT of KA versus MA in a study of 41 patients undergoing bilateral TKA with one side having KA and the other MA [25]. In this study, the clinical outcomes were similar. Twenty patients had no preference between the two techniques while 14 of the remaining 21 preferred the knee with KA. Although this information is interesting, it is inappropriate to include data from a study with bilateral knee replacements performed with two techniques in the same patient into a meta‐analysis comparing unilateral TKA with one of two different techniques on any given patient.

Finally, two studies that have been widely included in meta‐analyses, those by Yeo and Matsumoto, did not define KA according to the widely accepted definition [24, 43]. In these studies, the authors applied population means for proximal tibial and distal femoral alignment to create a different definition of KA where all patients are corrected to the population average alignment, rather than following the true definition of KA which is to customise the alignment to that specific patient. According to Matsumoto, “a 3‐degree varus was chosen based on the report of an inclination of the tibial plateau of about 3 degrees in asymptomatic volunteers regardless of age”. Yeo along the same lines, indicates that in “the KA group, the tibial cut was planned at 2° of varus to the mechanical axis of the tibia while the femoral cut was planned at 2° of valgus to the mechanical axis”. Since the publication of these papers, the erroneous definition of KA has been addressed in letters to the editor [13, 29]. Unfortunately, despite the recognition of these by the respective journals, these studies have been included in a number of systematic reviews and meta‐analyses as shown here, compromising the conclusions of those reviews.

**Figure 2:**
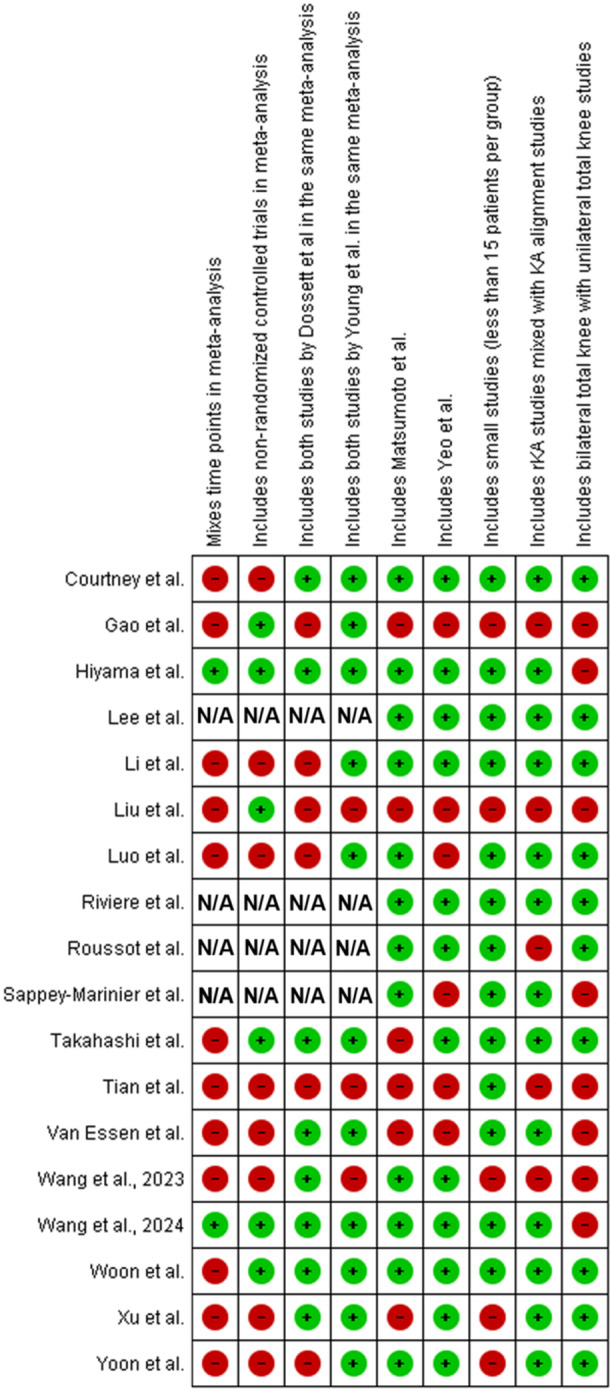
Technical problems of included systematic reviews/meta‐analyses of RCTs comparing KA to MA in TKA potentially compromising conclusions of the studies.

Finally, studies that included both RCTs and nonrandomized trials in the same meta‐analysis were noted as this methodology increases the risk of bias in their conclusions. A summary of the technical concerns in this group of studies is presented in graphical format in Figure 2.

**Figure 3:**
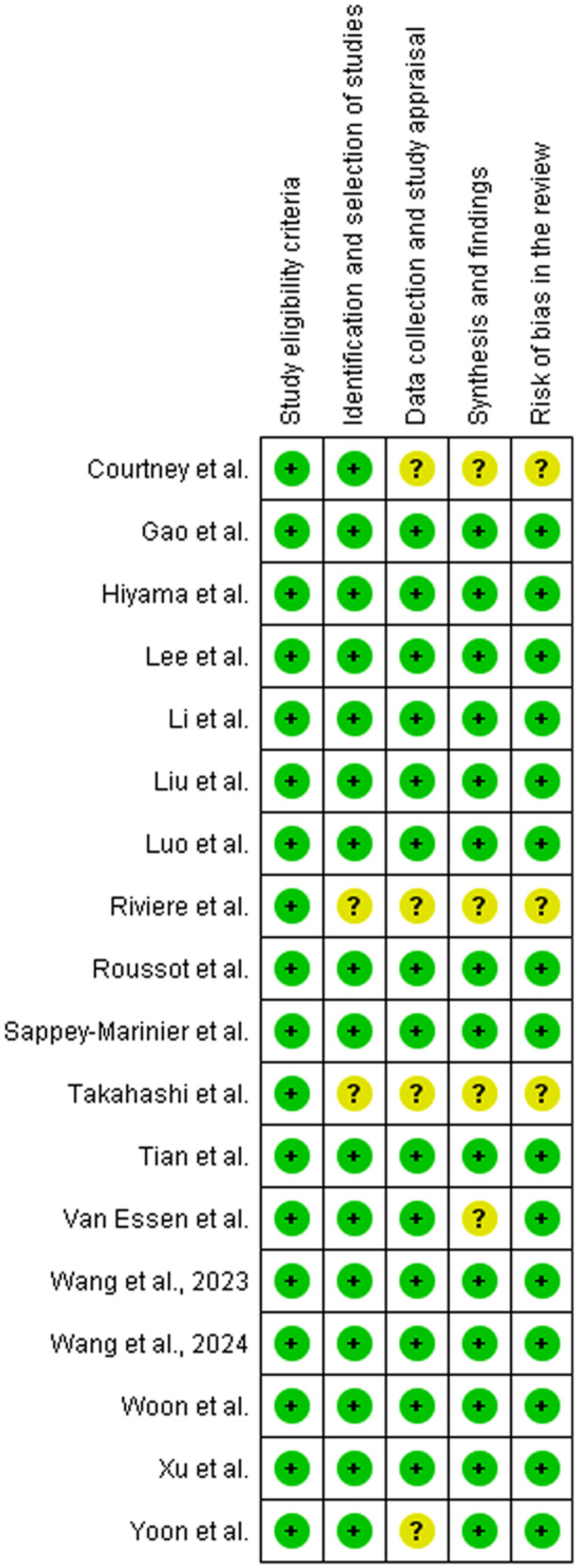
Risk of Bias in included systematic reviews/meta‐analyses of RCTs comparing KA to MA in TKA.

Risk of Bias according to the ROBIS tool demonstrated generally low risk and robust methodology for study inclusion [40]. The Risk of Bias analysis is presented in Figure 3.

## DISCUSSION

The most important finding of this study was that there are multiple technical issues among the systematic reviews and meta‐analyses of KA versus MA for TKA. These issues limited the conclusions that can be drawn from this body of literature. Placing a knee replacement in the identical spatial position as the native knee, as described in the kinematic alignment technique, should theoretically lead the ligaments to be under physiological tension throughout the ranges of motion. Thus, a kinematically aligned knee replacement should presumably lead to higher patient satisfaction. To prove these advantages clinically, a multitude of case series and randomised controlled trials have been performed.

Many of these randomised controlled trials have consisted of small numbers of patients or have had other methodological issues which we have previously described [34]. This has limited the conclusions that could be drawn from these studies in isolation. In an effort to address these shortcomings, a number of systematic reviews and meta‐analyses have been published on the topic. The objective of the current study is to assess these reviews, not only from the perspective of statistical rigour, but also based on technical considerations indicating that the author's understanding of the studies they included in their systematic review and meta‐analyses. These technical considerations would include factors such as attention to the time point of follow‐up, avoidance of the same patients twice in the same meta‐analysis, exclusion of very small studies, comingling patients with bilateral knee replacements with those with unilateral knee replacements in the same analysis, avoiding including rKA and KA in the same analysis, and understanding the technical definitions of KA and MA. Our findings indicated that many of these reviews demonstrated methodological flaws as detailed in Figure 2. From a statistical point of view, the studies were well designed and generally demonstrated a low risk of bias as shown in Figure 3.

There are incentives for researchers to expand the numbers included in their meta‐analyses to achieve higher confidence in the conclusions. This interest must be balanced with the need to include uniform groups of patients in the analysis. The systematic review and meta‐analysis literature comparing mechanical alignment to kinematic alignment for total knee replacement highlights many such cases where some authors have over‐extended the numbers of patients to include in their reviews.

For example, the technique of restricted kinematic alignment has been developed as a method of finding a balance between the basic tenets of kinematic alignment, namely maintaining the patient's native alignment after the knee replacement, while also avoiding the extremes of such a philosophy, such as maintaining an outlier alignment of the native knee after knee replacement. Although the rKA technique has merits as does the KA technique, one can easily see that these two philosophies have substantial differences and should not be mixed in the same metaanalysis.

In another study, patients were treated with a kinematically aligned knee replacement on one side and a mechanically aligned knee replacement on the other [25]. Although this is a valid study design, internally controlling the patient's demographic, psychometric, and physical attributes between the two interventions. The issue arises when patients from this study are mixed in a meta‐analysis with patients who have had unilateral knee replacement using one of the two techniques (MA or KA). The patient populations who undergo bilateral knee replacements have a uniquely different experience than those who have unilateral knee replacements and comparing them within the same meta‐analysis would be unjustified.

Finally, a number of studies incorrectly defined kinematic alignment. Kinematic alignment is an alignment technique that strives to maintain the native spatial positions of the distal femur and proximal tibia in such a way that the soft‐tissue envelope of a replaced knee sees the exact same tensions as that of the prearthritic native knee throughout the arc of motion. In this way, it is a customised technique where each patient has his or her own individual unique implant position based on their anatomy. In two publications, the authors deviated from this definition of kinematic alignment and had every knee within their KA groups aligned to a population mean of tibiofemoral alignment. Although, this may lead to good clinical results, it is not an individualised treatment, and it does not constitute kinematic alignment according to the widely accepted definition. The inclusion of these two studies within multiple systematic reviews and meta‐analyses was yet another factor compromizing the conclusions of those reviews.

## CONCLUSION

In summary, the currently available systematic reviews and meta‐analyses comparing KA to MA for TKA have limited capacity to show that one technique is superior to the other. Surgeons are using increasingly accurate and precise tools including computer‐assisted navigation and robotic technology to implement their alignment goals while still not having an answer as to the optimal target.

Our hope from this research is that larger randomised controlled trials with clear definitions for each alignment method can be developed. Computer‐assisted technology will facilitate this research in providing clear quantitative information as to the final alignment achieved at surgery.

## AUTHORS CONTRIBUTIONS

All authors contributed to the data acquisition, synthesis, and writing of the manuscript.

## CONFLICT OF INTEREST STATEMENT

None of the authors have any conflicts of interest related to this manuscript.

## ETHICS STATEMENT

As a systematic review of previous systematic reviews, this study does not require committee approval. The study was performed in accordance with ethical guidelines as spelled out in the 1964 Helsinki Declaration and its later amendments.

## Data Availability

All data used in this manuscript is publicly available on the utilised databases.
